# Extension of a gaseous dry deposition algorithm to oxidized volatile organic compounds and hydrogen cyanide for application in chemistry transport models

**DOI:** 10.5194/gmd-14-5093-2021

**Published:** 2021-08-16

**Authors:** Zhiyong Wu, Leiming Zhang, John T. Walker, Paul A. Makar, Judith A. Perlinger, Xuemei Wang

**Affiliations:** 1 Air Quality Research Division, Science and Technology Branch, Environment and Climate Change Canada, Toronto, ON, M3H 5T4, Canada; 2 ORISE Fellow at the US Environmental Protection Agency, Center for Environmental Measurement and Modeling, Research Triangle Park, NC 27711, USA; 3 US Environmental Protection Agency, Center for Environmental Measurement and Modeling, Research Triangle Park, NC 27711, USA; 4 Civil and Environmental Engineering Department, Michigan Technological University, Houghton, MI 49931, USA; 5 Institute for Environmental and Climate Research, Jinan University, Guangzhou, 510632, China

## Abstract

The dry deposition process refers to flux loss of an atmospheric pollutant due to uptake of the pollutant by the Earth’s surfaces, including vegetation, underlying soil, and any other surface types. In chemistry transport models (CTMs), the dry deposition flux of a chemical species is typically calculated as the product of its surface layer concentration and its dry deposition velocity (*V*_d_); the latter is a variable that needs to be highly empirically parameterized due to too many meteorological, biological, and chemical factors affecting this process. The gaseous dry deposition scheme of Zhang et al. (2003) parameterizes *V*_d_ for 31 inorganic and organic gaseous species. The present study extends the scheme of Zhang et al. (2003) to include an additional 12 oxidized volatile organic compounds (oVOCs) and hydrogen cyanide (HCN), while keeping the original model structure and formulas, to meet the demand of CTMs with increasing complexity. Model parameters for these additional chemical species are empirically chosen based on their physicochemical properties, namely the effective Henry’s law constants and oxidizing capacities. Modeled *V*_d_ values are compared against field flux measurements over a mixed forest in the southeastern US during June 2013. The model captures the basic features of the diel cycles of the observed *V*_d_. Modeled *V*_d_ values are comparable to the measurements for most of the oVOCs at night. However, modeled *V*_d_ values are mostly around 1 cm s^−1^ during daytime, which is much smaller than the observed daytime maxima of 2–5 cm s^−1^. Analysis of the individual resistance terms and uptake pathways suggests that flux divergence due to fast atmospheric chemical reactions near the canopy was likely the main cause of the large model–measurement discrepancies during daytime. The extended dry deposition scheme likely provides conservative *V*_d_ values for many oVOCs. While higher *V*_d_ values and bidirectional fluxes can be simulated by coupling key atmospheric chemical processes into the dry deposition scheme, we suggest that more experimental evidence of high oVOC *V*_d_ values at additional sites is required to confirm the broader applicability of the high values studied here. The underlying processes leading to high measured oVOC *V*_d_ values require further investigation.

## 1 Introduction

Atmospheric pollutants impact human health and can also cause detrimental effects on sensitive ecosystems (Wright et al., 2018). Quantifying atmospheric deposition for atmospheric pollutants is needed to estimate their lifetimes in air and deposition rates to ecosystems. In the mass continuity equation of a chemistry transport model (CTM), atmospheric deposition is calculated separately for dry and wet deposition fluxes. Dry deposition refers to the removal process through which pollutants are taken up by the Earth’s surface, and this process, while being quite slow, is a continuous process happening all the time, even during precipitation. In contrast, wet deposition is fast but episodic, and pollutants need to first be incorporated into hydrometeors before being delivered to the surface via precipitation. The amount of dry deposition of a pollutant of interest is typically calculated as the product of its ambient concentration and its dry deposition velocity (*V*_d_), with *V*_d_ being calculated using empirically developed dry deposition schemes (Wesely and Hicks, 2000). In most *V*_d_ formulations, turbulent and diffusion effects are parameterized as aerodynamic and quasi-laminar resistance, respectively, above and sometimes also inside the canopy. Uptake effects by canopies, underlying soils, and any other surface types are parameterized as canopy (or surface) resistance, which includes several flux pathways such as to stomatal, cuticle, and soil. All of these flux pathways can be simultaneously affected by meteorological, biological, and chemical factors, most of which cannot be explicitly considered and are thus highly empirically parameterized in existing dry deposition schemes, which are known to have large uncertainties even for the most commonly studied chemical species such as O_3_, SO_2_, and more commonly measured nitrogen species with relatively rich flux datasets (Flechard et al., 2011; Wu et al., 2012, 2018)

Existing dry deposition schemes have thus far considered a small number of oxidized volatile organic compounds (oVOCs). Due to the lack of field flux data for oVOCs, *V*_d_ of these species is typically parameterized based on physicochemical properties, taking SO_2_ and O_3_ as references (Wesely, 1989; Zhang et al., 2003). In these existing schemes, *V*_d_ values of most oVOCs are of a similar order of magnitude as or slightly smaller than that of *V*_d_ of O_3_. However, higher daytime *V*_d_ values for certain oVOCs than predicted by these schemes were recently reported by two studies (Karl et al., 2010; Nguyen et al., 2015). In one study Karl et al. (2010) found that *V*_d_ values of oVOCs calculated using existing schemes are about a factor of 2 lower than those based on canopy-level concentration gradient measurements over six forest and shrubland sites. *V*_d_ in their study was calculated from an inverse Lagrangian transport model with concentration gradient data as model input. The ratios of magnitudes between *V*_d_(oVOCs) and *V*_d_(O_3_) in the study of Karl et al. (2010) are similar to those of Zhang et al. (2003) in that *V*_d_(oVOCs) are slightly smaller than *V*_d_(O_3_) in both cases. However, the typical daytime *V*_d_(O_3_) over vegetated canopies is around 1 cm s^−1^ in the literature from numerous studies (see summary in Silva and Heald, 2018), and the value in Karl et al. (2010) is much higher (e.g., up to 2.4 cm s^−1^ at canopy top). One hypothesis explaining both high *V*_d_(O_3_) and high *V*_d_(oVOCs) would be the reaction of O_3_ with oVOCs, which depends on the chemical structure of the oVOC, but data required for validating this hypothesis are still lacking. We thus suspect that the very high *V*_d_(oVOCs) values presented in Karl et al. (2010) were likely caused by atmospheric chemical processes not typically considered in the dry deposition process. High *V*_d_(oVOCs) values were also observed over a temperate mixed forest in the southeastern US in a more recent short-term study (Nguyen et al., 2015), which again were suspected to be caused by atmospheric chemical reactions near vegetation surface. The flux measurements themselves also contain uncertainty. For example, Wu et al. (2015) showed that different measurement methods (e.g., flux gradient versus eddy correlation) resulted in very different daytime *V*_d_(O_3_) over the same forest canopy.

Hydrogen cyanide (HCN) is one of the most abundant cyanides present in the atmosphere (Singh et al., 2003) and is considered a biomass burning marker (Bunkan et al., 2013), but few existing studies have considered its dry deposition, which is critical to estimating the total sinks and atmospheric lifetimes of cyanides.

To meet the demands of modeling a large number of organic compounds in CTMs (Kelly et al., 2019; Moussa et al., 2016; Paulot et al., 2018; Pye et al., 2015; Xie et al., 2013), existing or newly developed air–surface exchange and dry deposition schemes need to be expanded to include additional oVOCs. At this stage with very limited knowledge of oVOC *V*_d_, air–surface exchange models based on various theoretical and/or measurement approaches should be developed so that these models can be made available to the scientific community where such models are urgently needed as well as for future evaluation and improvement should more flux measurements become available. For example, Nguyen et al. (2015) modified the Wesely (1989) scheme to fit the flux data. A more sophisticated model, with a bottom-up approach, was adopted in Nizzetto and Perlinger (2012) to handle air–canopy exchange of semivolatile organic compounds.

The original dry deposition scheme of Zhang et al. (2003) includes 9 inorganic species and 22 organic species. Most of these 22 organic species are oVOCs formed from oxidation of nonmethane hydrocarbons. To take advantage of the recent flux dataset of a large number of oVOCs and HCN collected over a temperate forest (Nguyen et al., 2015), the present study extends the Zhang et al. (2003) scheme by including 12 additional oVOC species and HCN while keeping the same original model structure and theory. These additional oVOCs include hydroxymethyl hydroperoxide, peroxyacetic acid, organic hydroxy nitrates, and other multifunctional species that are mainly formed from the oxidation of biogenic VOCs (e.g., isoprene and monoterpenes). Model parameters for these newly included species are theoretically constrained based on the effective Henry’s law constants and oxidizing capacities of the individual species as well as by considering the measured *V*_d_ values. Such an approach provides a top-down determination of *V*_d_ through comparison with measured (bottom-up) fluxes. Model–measurement comparison is conducted for *V*_d_ as well as resistance components and uptake pathways, results from which identify the major causes of model–measurement discrepancies. This study provides a computer code that is potentially useful for CTMs handling these oVOCs.

## 2 Methodology

### 2.1 Brief description of the *V*_d_ formulation

In the scheme of Zhang et al. (2003), *V*_d_ is calculated as follows:

(1) $$V_{\text{d}}\left(z\right)={\left(R_{\text{a}}\left(z\right)+R_{\text{b}}+R_{\text{c}}\right)}^{-1},$$

where *R*_a_ is the aerodynamic resistance, *R*_b_ the quasi-laminar sub-layer resistance, *R*_c_ the surface resistance, and *z* the reference height above the vegetation. *R*_c_ is parameterized as

(2) $$\frac{1}{R_{\text{c}}}=\frac{1-W_{\text{st}}}{R_{\text{s}}+R_{\text{m}}}+\frac{1}{R_{\text{ns}}},$$

(3) $$\frac{1}{R_{\text{ns}}}=\frac{1}{R_{\text{ac}}+R_{\text{g}}}+\frac{1}{R_{\text{cut}}},$$

where *R*_s_ is the canopy stomatal resistance, *R*_m_ the mesophyll resistance, *R*_ns_ the non-stomatal resistance including resistance for uptake by leaf cuticles (*R*_cut_) and by soil or ground litter (*R*_g_), *R*_ac_ in-canopy aerodynamic resistance, and *W*_st_ the fraction of stomatal blocking under wet conditions.

*R*_s_ is calculated as follows:

(4) $$\frac{1}{R_{\text{s,i}}}=G_{\text{s}}\left(\text{PAR}\right)f\left(\text{T}\right)f\left(\text{D}\right)f\left(\Psi \right)\frac{D_{\text{i}}}{D_{{\text{H}}_{\text{2}}\text{O}}}$$

Here *G*_s_(PAR) is the unstressed canopy stomatal conductance for water vapor, a function of photosynthetically active radiation (PAR). The dimensionless functions *f(T)*, *f(D)*, and *f(ψ)* range from 0 to 1, representing the fractional degree of stomatal closure caused by the stress from temperature, water vapor pressure deficit, and leaf water potential, respectively. $D_{{\text{H}}_{\text{2}}\text{O}}$ and *D*_i_ are the molecular diffusivities for water vapor and the gas of interest, respectively.

*R*_cut_ and *R*_g_ for any chemical species are scaled to those of SO_2_ and O_3_ with two species (*i*)-dependent scaling parameters *α*(*i*) and *β*(*i*):

(5) $$\frac{1}{R_{\text{cut}/\text{g}}\left(i\right)}=\frac{\alpha \left(i\right)}{R_{\text{cut}/\text{g}}\left({\text{SO}}_{2}\right)}+\frac{\beta \left(i\right)}{R_{\text{cut}/\text{g}}\left({\text{O}}_{3}\right)}.$$

Details of the *R*_s_-related formulas were described in Zhang et al. (2002), *R*_ns_-related formulas in Zhang et al. (2003), and *R*_a_ and *R*_b_ formulas in Wu et al. (2018).

### 2.2 Extension of the scheme to additional chemical species

Dry deposition of a gaseous compound to most canopy types is mainly through non-stomatal uptake during nighttime and through both non-stomatal and stomatal uptake during daytime. The non-stomatal uptake depends on water solubility and reactivity of the species, which can be quantified by its effective Henry’s law constant (H*) and oxidizing capacity, respectively (Wesely, 1989; Zhang et al., 2002).

In the Supplement, Table S1 lists H* values and Table S2 lists the oxidizing capacities for oVOCs and HCN considered in the present study. As shown in Eq. (5) above, two model parameters (*α* and *β*) are needed for every chemical species to calculate the non-stomatal uptake, with *α* being dependent on H* and *β* dependent on oxidizing capacity. Initial *α* values were first given based on the relative magnitudes of H* of all the chemical species and that of SO_2_. Considering that the majority of the chemical species are very reactive, a value of 1.0 was used for *β* for most species and smaller values for a few less reactive species. *α* and *β* values were then adjusted based on the agreement of nighttime *V*_d_ between modeled values and measured fluxes obtained from a forest site in the southeastern US during summer (Nguyen et al., 2015). When adjusting *α* and *β* values, two rules were first applied: (1) the trends in *α* (or *β*) values between different chemical species should be consistent with the trends of their log(H*) (or oxidizing capacity) (see Fig. S1 in the Supplement for the finalized *α* versus log(H*)), and (2) modeled mean and median nighttime *V*_d_ should be mostly within a factor of 2.0 of the measured values (see discussion in Sect. 3.2 below). Only after these two rules were satisfied were the possible maximum *α* and *β* values chosen to reduce the gap between the modeled and measured daytime *V*_d_, knowing that model-predicted *V*_d_ values were mostly lower than the measured ones. The finalized *α* and *β* values for the additional 12 oVOCs and HCN are listed in Table 1.

Model parameters chosen for the additional oVOCs and HCN can produce the magnitude of nighttime *V*_d_ for nearly all the chemical species, but they inevitably underpredicted daytime *V*_d_ for several oVOC species with very high measured daytime *V*_d_ values. We designed the model parameters this way due to the following considerations: (1) some of the chemical processes causing flux loss at the surfaces may be treated separately in the mass continuity equation in chemical transport models, (2) some of the oVOCs may also experience bidirectional air–surface exchange, and (3) more flux measurements are needed to confirm if the very high daytime flux for certain oVOCs is a universal phenomenon, noting that the existing data used here were from a short period of several days and over only one surface type.

Besides *α* and *β*, another chemical-species-dependent parameter that needs to be arbitrarily chosen is *R*_m_. *R*_m_ for HCN was set to 100sm^−1^ based on its effective Henry’s law constants and oxidizing capacities. Karl et al. (2010) found that enzymatic conversion can be an efficient pathway for the immobilization of oVOCs (e.g., methacrolein and methyl vinyl ketone, acetaldehyde, methacrolein) within the leaf interior, besides dissolution and oxidation, which suggests that the magnitude of *R*_m_ for oVOCs is minimal. Thus, the *R*_m_ for the oVOCs was set to 0sm^−1^ (Table 1).

### 2.3 Field flux data

The fluxes of 16 atmospheric compounds (including 13 oVOC species, HCN, hydrogen peroxide – H_2_O_2_, and nitric acid – HNO_3_) were measured using the eddy covariance (EC) technique at the Centreville (“CTR”) Southeastern Aerosol Research and Characterization Study (SEARCH) site (hereinafter referred to as CTR). Note that formic acid (HCOOH) is the only overlapping oVOC species between the original Zhang et al. (2003) scheme and the flux measurement dataset. The CTR site (Brent, Alabama; 32.90° N, 87.25° W) is surrounded by a grassy field to the south and a temperate mixed forest that is part of the Talladega National Forest in all the other directions. The forest canopy is comprised of needleleaf coniferous (shortleaf, longleaf, and loblolly pine; ∼40%) and broadleaf deciduous (primarily oak, sweetgum, and hickory; ∼60%) tree species. The canopy height near the tower is on average ∼10m with a leaf area index (LAI) of ∼4.7m^2^ m^−2^. A 20m metal walk-up tower is used as the main structure supporting instruments that measured the eddy covariance fluxes and related meteorological variables. The sonic anemometer and the gas inlet were mounted at a height of about 22m, facing north toward the forest. Mixing ratios of gas-phase compounds were measured with negative-ion chemical ionization mass spectrometry (CIMS) at 8Hz or faster. A database of half-hourly *V*_d_ for 16 atmospheric compounds covering 5 non-continuous days in June 2013 was obtained at the site. During these periods, the predominant winds were northerly, which is ideal to sample air from the forest (Fig. S2), and the requirement for energy balance closure was met (see Nguyen et al., 2015). At CTR, it was typically humid (RH 50%–80%) and warm (28–30°C) in the daytime during the experiment (Fig. S3). A comprehensive description of the *V*_d_ dataset, data processing protocols, instrumental methods, uncertainty analysis, and site characterizations can be found in Nguyen et al. (2015).

## 3 Results and discussion

### 3.1 Comparison of modeled resistance components

#### 3.1.1 Atmospheric resistances (*R*_a_ and *R*_b_)

For very reactive and soluble substances such as HNO_3_ and H_2_O_2_, *R*_c_ is often assumed to be close to 0 (Hall and Claiborn, 1997; Meyers et al., 1989; Valverde-Canossa et al., 2006; Wesely and Hicks, 2000). The analysis of the measurement data showed that the daytime averaged *V*_d_ for HNO_3_ and H_2_O_2_ fit the rate of deposition well without surface resistance ($V_{d}=1/\left[R_{\text{a}}+R_{\text{b}}\right]$) (Nguyen et al., 2015), which supports the assumption of near-zero *R*_c_ for HNO_3_ and H_2_O_2_ over the mixed deciduous–coniferous CTR site under a humid environment. Therefore, the measured *V*_d_ of HNO_3_ and H_2_O_2_ can be used to evaluate the modeled atmospheric resistances for those species (the sum of *R*_a_ and *R*_b_). *R*_a_ represents the resistance for turbulent transport between the reference height and the surface and is not chemical-compound-specific. *R*_b_ quantifies the resistance for the mass transfer across the thin layer of air in contact with surface elements and is a function of the molecular diffusivity of a specific compound (Wesely and Hicks, 1977). In theory, the differences in *R*_b_ between any two gaseous species are only determined by differences in their molecular diffusivity at any given turbulent condition.

**Figure 1.**
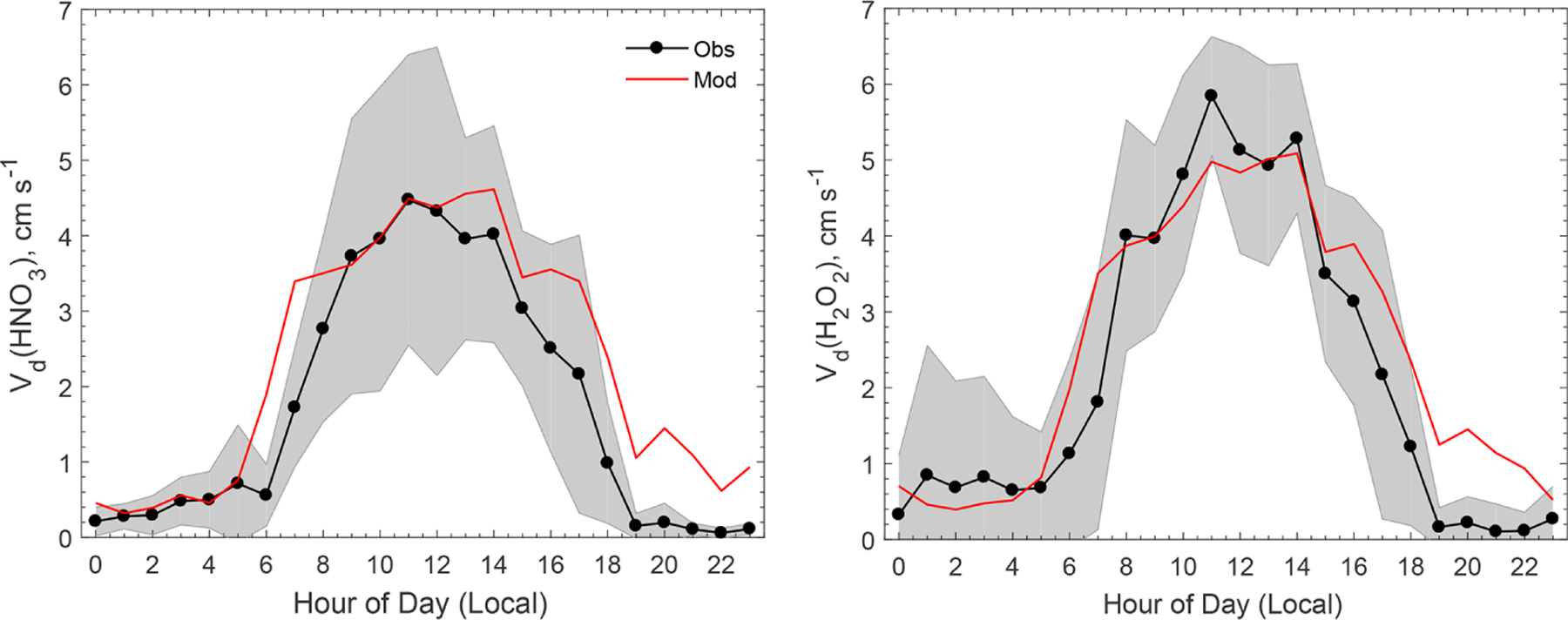
Comparison of the observed and modeled average diel variations of dry deposition velocities (*V*_d_) for HNO_3_ and H_2_O_2_. The shaded area indicates the standard deviation of the observations. The model assumes that surface resistances (*R*_c_) for HNO_3_ and H_2_O_2_ are zero.

Figure 1 compares the modeled average diel variations of *V*_d_ for HNO_3_ and H_2_O_2_ against observations. The measured *V*_d_ values for HNO_3_ and H_2_O_2_ peaked around noon at about 4 and 6 cm s^−1^, respectively, and were less than 1 cm s^−1^ during the night. The model reproduced the diel pattern and captured the peak *V*_d_ values at noon well. During the early nighttime (hours 19–23), the modeled *V*_d_ values for HNO_3_ and H_2_O_2_ were on the order of 1 cm s^−1^, which is much higher than the measurements (*<*0.2 cm s^−1^). During the night, *R*_a_ dominates atmospheric resistance as it is usually much larger than *R*_b_ in magnitude. This discrepancy between the measurement and the model during the early night could be due to the stability correction functions used in the *R*_a_ calculation (the equations can be found in the article by Wu et al., 2018), which is subject to large uncertainties under nocturnal stable conditions (Högström, 1988). The measurements indicated that H_2_O_2_ deposited slightly faster than HNO_3_, and the model reproduces this well, as shown in Fig. 1. Modeled *R*_b_ for H_2_O_2_ is always smaller than that for HNO_3_ due to the smaller molecular weight and the larger molecular diffusivity. Overall, the model was in good agreement with the measurements regarding *V*_d_ for HNO_3_ and H_2_O_2_, implying that the parameterization for atmospheric resistances (*R*_a_ and *R*_b_) was reasonable for the site during the study period.

#### 3.1.2 Stomatal resistance (*R*_s_)

**Figure 2.**
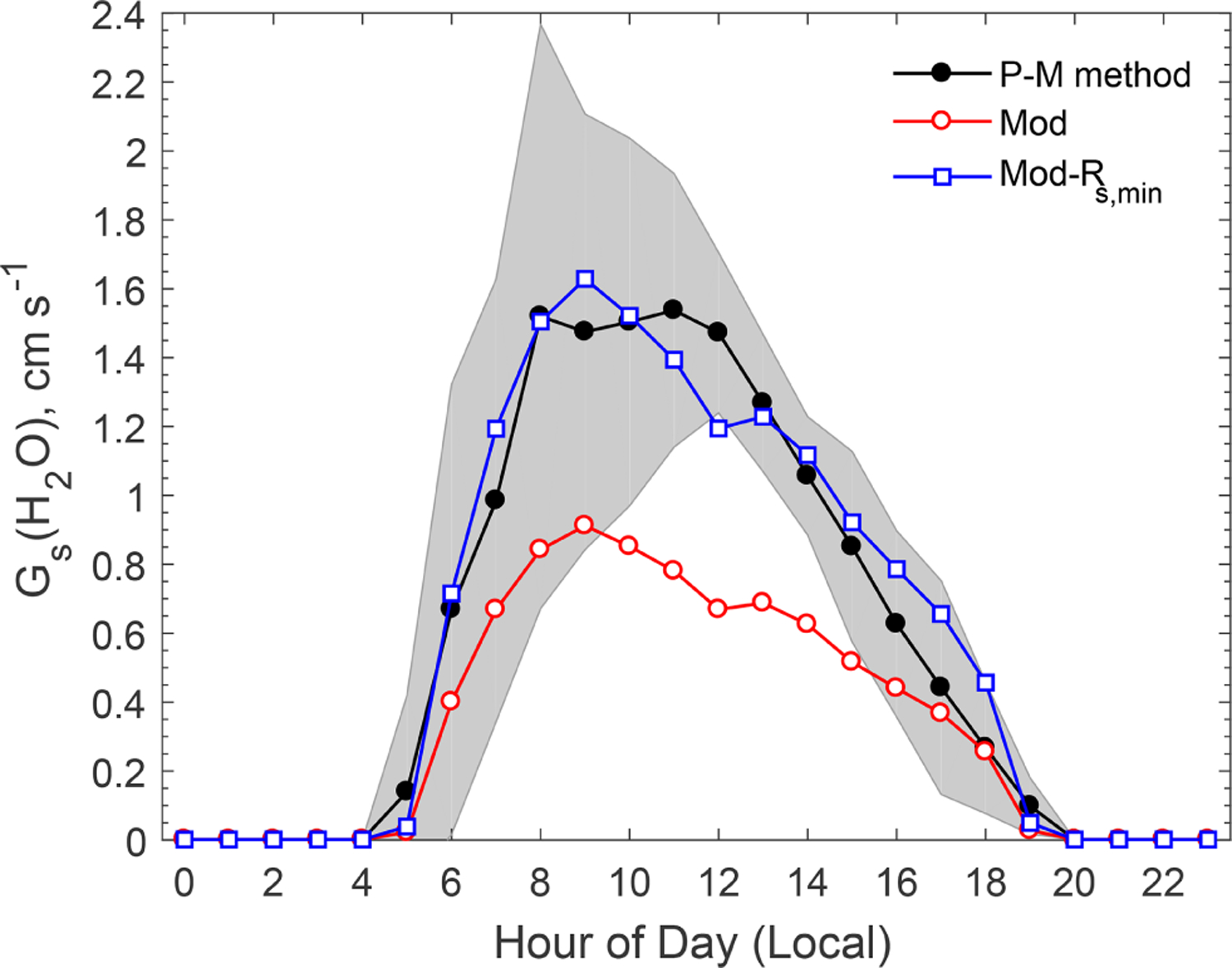
Comparison of observation-based and modeled average diel variations of stomatal conductance (*G*_s_) for water vapor. The shaded area indicates the standard deviation of the observation-based *G*_s_(H_2_O) estimated by the P-M method. “Mod-*R*_s,min_” refers to a model sensitivity test in which *R*_s,min_ was reduced by 40%.

Over vegetated areas, gas molecules can exit and enter the leaf through the stomata by molecular diffusion, similar to the leaf–air exchange of water vapor and CO_2_. In dry deposition models, *R*_s_ for water vapor is estimated using evapotranspiration stomatal submodels, an approach that is also popular in the land surface and climate communities. *R*_s_ is extended to any gas species using the ratio of molecular diffusivity of the species of interest to that of water vapor (Pleim and Ran, 2011; Wesely and Hicks, 2000). Figure 2 compares the modeled canopy stomatal conductance (*G*_s_ = 1*/R*_s_) for water vapor against the observation-based estimates. The observation-based *G*_s_ was estimated by using the inversion of the Penman–Monteith (P-M) equation (Monteith and Unsworth, 1990), which calculates *R*_s_ for water vapor by using measured water vapor fluxes and related meteorological data (e.g., humidity, temperature). The evaporation from soil water and liquid water on the vegetation surfaces is usually a minor contribution to the total water vapor flux observed above a forest canopy during daytime in summer. It was assumed that 85% of the water vapor flux originated from transpiration in this study, following that used in the study of Turnipseed et al. (2006) at Duke Forest, North Carolina. Note that a value of 90% was used by Clifton et al. (2017) at Harvard Forest, Massachusetts. The uncertainty of the calculated *R*_s_ related to the uncertainty in water vapor flux portion (on an order of 10%) is much smaller than the differences between the modeled and the observation-based stomatal conductance (by a factor of 2) as discussed below.

As shown in Fig. 2, the model reproduced the basic diel pattern in *G*_s_ (i.e., highest values between 08:00 and 11:00), but the peak value is only about half of the observation-based values. The Jarvis-type stomatal submodel (Jarvis, 1976) is known for its linear dependence on the prescribed minimum stomatal resistance (*R*_s,min_), a term that is subject to large uncertainties (Kumar et al., 2011; Wu et al., 2018, 2011). A series of tests conducted by iteratively adjusting the *R*_s,min_ values showed the modeled *G*_s_ to be in better agreement with observations if *R*_s,min_ was decreased by 40% (Fig. 2). Modeled *G*_s_ with the adjusted *R*_s,min_ was in good agreement with the observation-based values most of the time, though the modeled values were slightly smaller than the observation-based estimates around noon. Analysis of the *R*_s_ parameterization indicates that this discrepancy was related to the stress function for water vapor pressure deficit (VPD) used in the Jarvis-type stomatal submodel, which may overpredict the stress on stomatal opening due to high VPD around noon.

#### 3.1.3 Non-stomatal resistance (*R*_ns_)

To assess if the non-stomatal resistance (*R*_ns_) parametrization (Eq. 3) is reasonable, modeled 1*/R*_ns_ (defined as *G*_ns_) values are compared with the non-stomatal portion of the flux, the inverse of which is termed the residual conductance (*G*_residual_). *G*_residual_ includes all processes influencing deposition aside from *R*_a_, *R*_b_, *R*_m_, and *R*_s_, calculated as ${\left[V_{\text{d}}^{-1}-\left(R_{\text{a}}+R_{\text{b}}\right)\right]}^{-1}-{\left(R_{\text{s}}+R_{\text{m}}\right)}^{-1}$. Here *V*_d_ is from the observations, *R*_a_ and *R*_b_ are calculated by the model driven by the observed meteorology, *R*_s_ is the observation-based estimate by the P-M method adjusted by the molecular diffusivity of each gas (similar to Eq. 4), and *R*_m_ is listed in Table 1. The uncertainties in individual resistance terms of Zhang et al. (2003) and several other dry deposition schemes have been thoroughly assessed by Wu et al. (2018), from which we believe *G*_residual_ estimated using the above formula is meaningful although with large uncertainties. The estimated *G*_residual_ can provide useful information on the flux*/V*_d_ resulting from processes such as deposition to the leaf cuticle and ground (i.e., non-stomatal) or chemical loss due to reactions within and near the canopy that lead to flux divergence.

**Figure 3.**
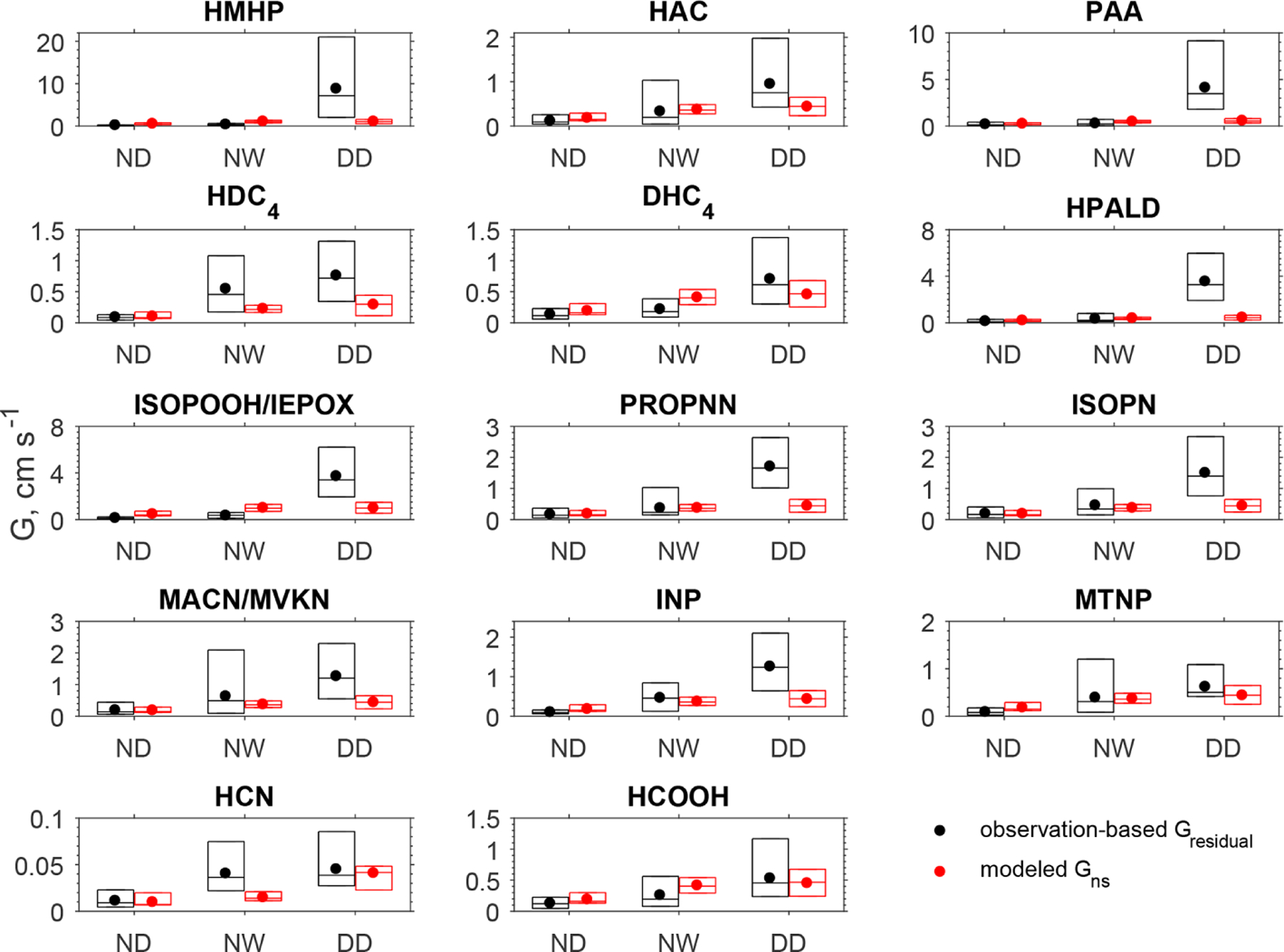
Box plot of the observation-based residual conductance (*G*_residual_) and the modeled non-stomatal conductance (*G*_ns_) during the nighttime dry period (ND, *n* = 88), nighttime wet period (NW, *n* = 40), and daytime dry period (DD, *n* = 85). In each box, the central mark is the median, and the edges of the box are the 25th and 75th percentiles. The filled dots represent the arithmetical mean of data between the 25th and 75th percentiles. Daytime is 09:00–17:00 (local time) and nighttime is 20:00–06:00 (local time). The wet surface conditions were determined in the model driven by the observations of relative humidity, precipitation rate, friction velocity, and temperature.

Figure 3 compares the observation-based *G*_residual_ for each oVOC species or HCN against the corresponding modeled non-stomatal conductance (*G*_ns_) under different conditions. The mean and median values are presented in Table S3. During the nighttime when the canopy surface was dry (no dew), the *G*_residual_ for oVOC species ranged from 0.08 to 0.18 cm s^−1^, and the modeled *G*_ns_ was comparable in magnitude. When the surface was wet from dew formation on leaves and needles, the oVOC species showed an increase in *G*_residual_ by 55%–440% compared to the nighttime dry surface. The model captured the increases in non-stomatal uptake when the surface become wet with dew, although it may underestimate (e.g., HDC_4_, INP, HCN) or overestimate (e.g., PAA, DHC_4_, HCOOH) the wetness effects. During the daytime of the study period, no precipitation was recorded at the CTR site (Fig. S3) and the canopy surface was dry. The mean *G*_residual_ for oVOCs ranged from 0.5 to 8.7 cm s^−1^ during the daytime, which is much higher than the modeled *G*_ns_ for most species (0.2–1 cm s^−1^). Figure S4 presents the diel variations of *G*_residual_ and *G*_ns_, and it shows that the modeled *G*_ns_ had smaller diel variations than those of *G*_residual_; large differences in magnitude can be seen during the daytime. The modeled *G*_ns_ showed a peak during the early morning (around 07:00), which may be due to the enhanced non-stomatal uptake by dew-wetted surfaces.

### 3.2 Evaluation of modeled deposition velocities

**Figure 4.**
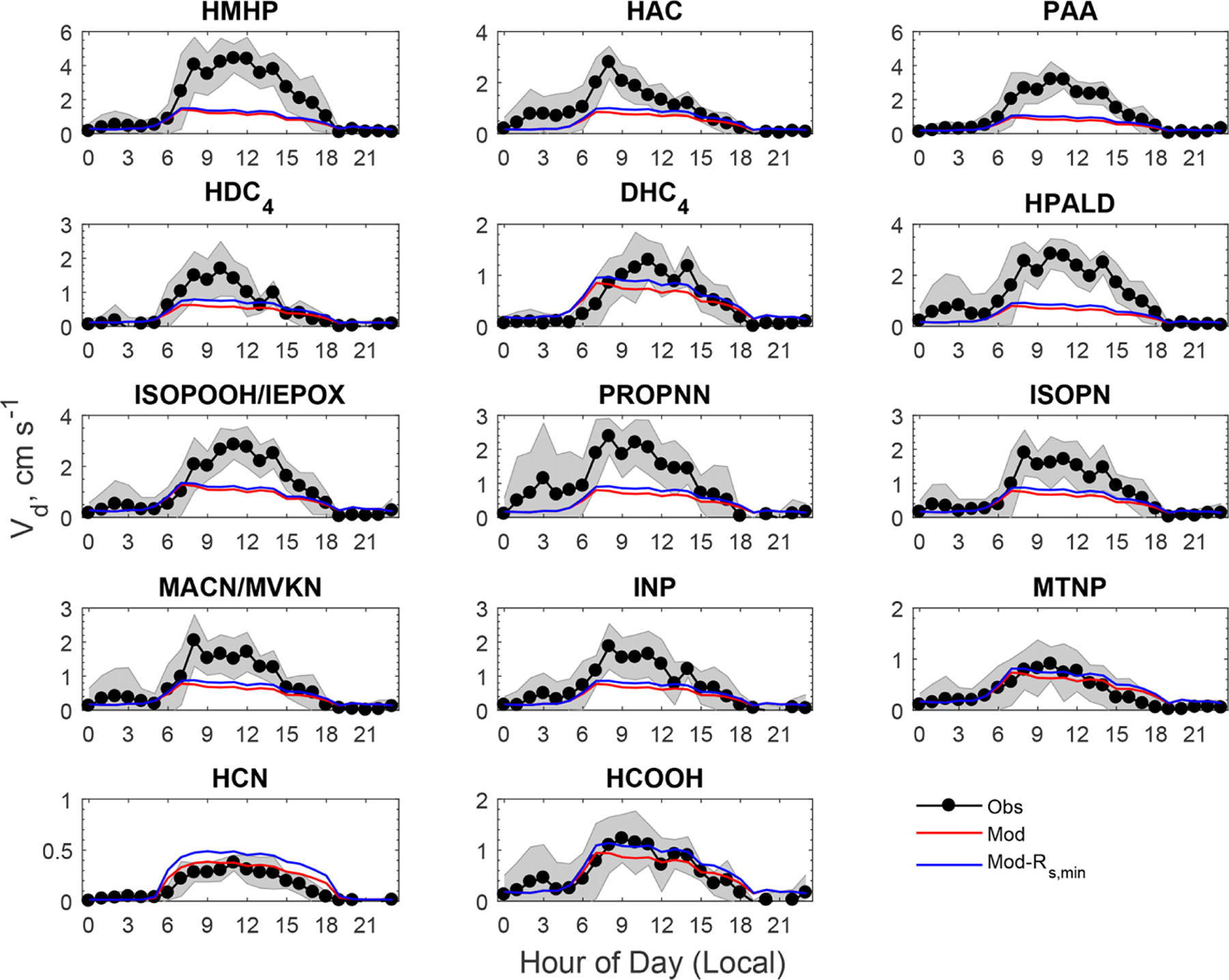
Comparison of averaged diel cycles of observed and modeled dry deposition velocities (*V*_d_) of oVOCs and HCN. The shaded area indicates the standard deviation of the observations. “Mod-*R*_s,min_” refers to a sensitivity test in which *R*_s,min_ was reduced by 40%.

Figure 4 shows a model–measurement comparison of diel *V*_d_ of the oVOCs and HCN, and Table 2 presents the statistical results of the comparison. As described in Sect. 2, the assigned *α* and *β* values should first produce reasonable nighttime *V*_d_. Modeled nighttime mean *V*_d_ values were very close to measurements for the majority of the chemical species, although the differences were somewhat larger for the median values (Table 2). Three species (HAC, HPALD, PROPNN) still had 50% lower modeled than measured nighttime mean *V*_d_ but had slightly higher modeled than measured nighttime median *V*_d_. In contrast, modeled daytime mean *V*_d_ values were more than 50% lower than the measured values for four species (HMHP, PAA, HPALD, ISOPOOH/IEPOX) and were also significantly lower for several other species. Only three species (MTNP, HCN, HCOOH) had comparable modeled and measured *V*_d_ for both daytime and nighttime. One species (DHC_4_) had slightly lower modeled than measured daytime mean or median *V*_d_, but with an opposite trend for nighttime *V*_d_.

**Figure 5.**
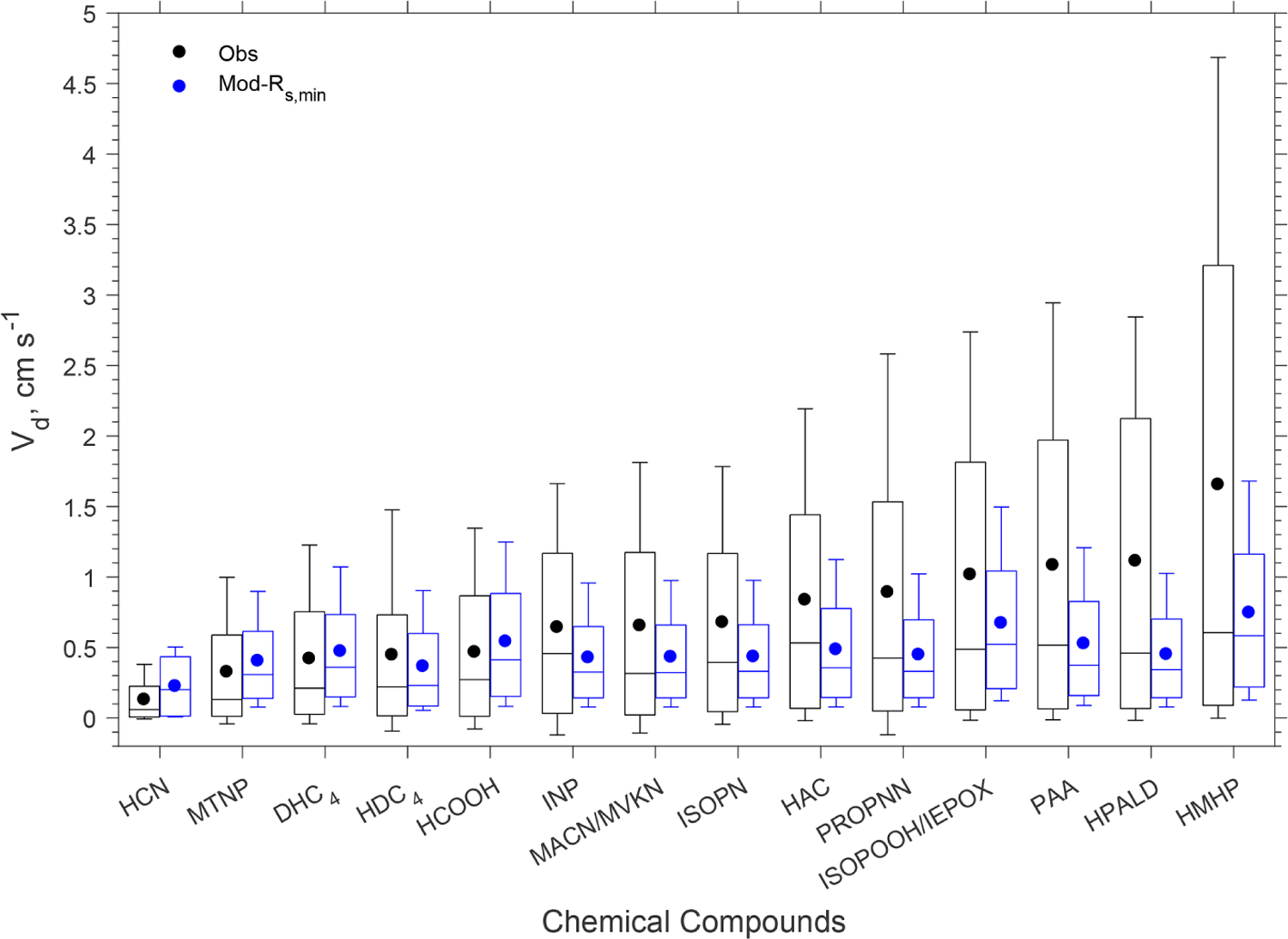
Box plot of observed and modeled hourly dry deposition velocities (*V*_d_) of oVOCs and HCN. In each box, the central mark is the median, the edges of the box are the 25th and 75th percentiles, and the whiskers extend to the 10th and 90th percentiles. The filled dots represent the arithmetical mean of all the data. “Mod-*R*_s,min_” refers to a sensitivity test in which *R*_s,min_ was reduced by 40%.

The model reproduced the basic features of the diurnal pattern of the observations, showing the highest values during the day and the lowest values at night. Correlation coefficients between the measurement and the model ranged from 0.52 to 0.77. At night, the measured *V*_d_ for the oVOCs remained relatively low, typically ranging from 0.1–0.5 cm s^−1^, and the model produced the same magnitudes for most of the species. During the daytime, the model can only capture the magnitudes of the measured *V*_d_ for a few species (e.g., HCN, HCOOH, MTNP, DHC_4_), the peak *V*_d_ values of which were less than 1.5 cm s^−1^. For the other species, the measured peak *V*_d_ values were in the range of 2 to 5 cm s^−1^, while the modeled results were below 1 cm s^−1^. As shown in Sect. 3.1.2, the modeled *G*_s_ was likely underestimated when compared to the simultaneous measurements of water vapor flux. Adjusting *G*_*s*_ higher by 67% (through reducing *R*_s,min_ by 40%) can only increase the modeled *V*_d_ of the oVOCs by 10%–40% during the daytime (see the sensitivity test in Fig. 4), and the peak values were still mostly below 1 cm s^−1^. Figure 5 shows that the model captured the differences in measured *V*_d_ for the oVOCs to some extent. The model–measurement agreements were good for species with the measured mean *V*_d_ below 0.5 cm s^−1^, above which the discrepancy increased. For the measurements, the mean values were significantly larger than the median values, especially for the fast-deposited species, indicating that the distribution of the measured *V*_d_ values skewed to the right (high values). The model has better agreement with the measurements by comparing the median versus mean values.

### 3.3 Fast chemical reactions as potential causes of the daytime model–measurement discrepancies

**Figure 6.**
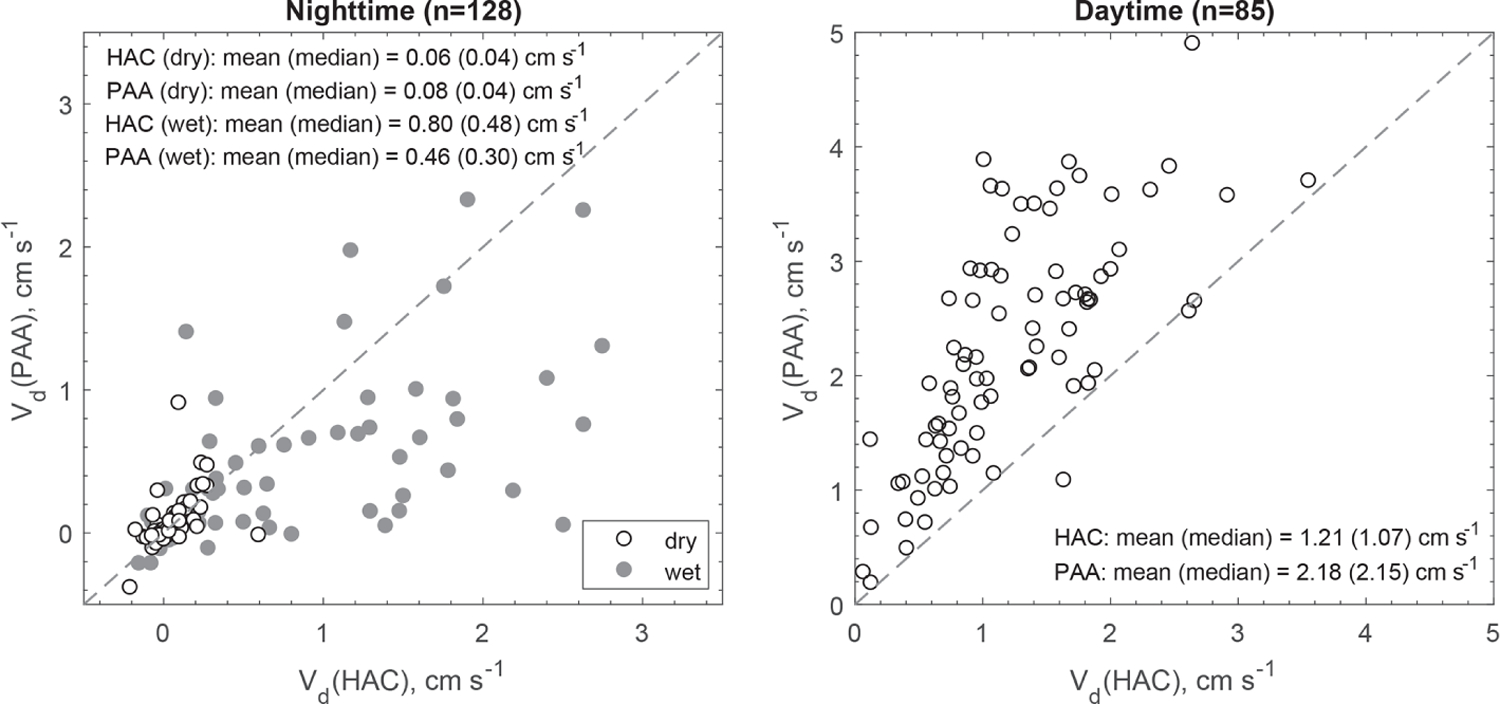
Scatter plot of the measured dry deposition velocities (*V*_d_) for hydroxyacetone (HAC) and peroxyacetic acid (PAA) during nighttime (20:00–06:00, local time) and daytime (09:00–17:00, local time). The shaded (white) cycles correspond to wet (dry) surface conditions.

At night when stomata are mostly closed and atmospheric chemical reactions are largely inhibited, the measured fluxes above the canopy should better represent non-stomatal surface uptake. In the presence of sunlight, fast chemical reactions between the inlet and canopy could make a significant or even dominant contribution to the measured fluxes of reactive species (Cape et al., 2009; Farmer and Cohen, 2008; Wolfe et al., 2011). The impact of fast chemical reactions on surface fluxes should be different for different chemical species. To verify this hypothesis, two chemical species (HAC and PAA) having similar molecular weights (74 and 76Da, respectively) but very different daytime fluxes were compared (Fig. 6). Their similar molecular diffusivities (controlled by molecular weight) suggest that they should be transferred through the quasi-laminar sub-layer and taken up through leaf stomata at similar rates, resulting in similar resistance components of *R*_b_ and *R*_s_. Note that *R*_a_ is universal to any trace gases and *R*_m_ is assumed to be negligible. Thus, the differences between their *V*_d_ should be caused by their different non-stomatal sinks. At night, *V*_d_ values were similar between HAC and PAA (median values: 0.04 cm s^−1^) over dry surfaces. When the surfaces were wet due to dew formation, *V*_d_ for both HAC and PAA increased (median values: 0.30–0.48 cm s^−1^). In contrast, *V*_d_(PAA) was much higher than *V*_d_(HAC) during daytime, suggesting additional or larger sinks for PAA compared to HAC. The reactivity parameters listed in Table S2 in the Supplement also suggest that PAA is more reactive than HAC. Thus, fast chemical processing and subsequent flux divergence above the canopy likely caused the large discrepancies between the measured and modeled *V*_d_ for the reactive oVOC compounds during the daytime.

Chemical processes can indeed cause flux divergence or convergence at the surface, which has been supported by growing evidence from field measurements (e.g., Farmer and Cohen, 2008; Min et al., 2014; Wolfe et al. 2009). For example, Wolfe et al. (2009) suggested that the differences in loss rate between the inlet and canopy may be an important contributor to the measured net flux of peroxyacetyl nitrate, irrespective of turbulent timescales. Photochemical OH production is reduced within canopies, which in turn slows down the oxidation of volatile organic compounds and the photolysis of organic nitrates. The oVOCs measured at the CTR site are mainly produced from the oxidation of isoprene and monoterpenes (Nguyen et al., 2015). Most of the oVOCs are quite chemically reactive and can undergo fast oxidation (e.g., multifunctional carbonyls), decomposition (e.g., HMHP), or photolysis (e.g., organic nitrates) (Müller et al., 2014; Nguyen et al., 2015). Vertical gradients in the chemical production and loss rates below the inlet can exhibit chemical flux divergence, which contributes to the net flux above the canopy. Quantifying the effects of chemical processing on the net flux would require a multi-layer model with resolved emission, deposition, turbulent diffusion, and chemical processes throughout the canopy, which is recommended for future studies (e.g., Ashworth et al., 2015; Bryan et al., 2012; Stroud et al., 2005; Wolfe and Thornton, 2011; Zhou et al., 2017).

Quantifying *V*_d_ as the ratio of flux to concentration at one measurement height only $\left(V_{\text{d}}=F/C_{zr}\right)$, rather than as the ratio of flux to the concentration difference at the measurement height and the surface $\left(V_{\text{d}}=F/\left[C_{zr}-C_{0}\right]\right)$, although commonly employed in analyzing eddy covariance flux measurements, is a simplification. It is valid for (1) matter that disappears nearly completely by reactions at the surface and (2) unstable or neutral conditions. Most chemical species considered here may satisfy the first condition. With regards to the second condition, our analysis is based on the assumption that, under stable conditions at nighttime, concentrations observed at the measurement height change in relation to the fluxes measured at this height. However, no relation between measured concentration and flux is typically observed due to the presence of a shallow stable boundary layer, connection between the stable free atmosphere and stable boundary layer by internal gravity waves, ground inversions, and low-level jets, leading to intermittent turbulence at the measurement height containing a gravity wave signal and non-steady-state conditions (Foken, 2017). Future efforts to model oVOC and HCN deposition velocities above forest canopies should be based on neutral or unstable boundary layer flux measurements only or, for example, on a modified Bowen ratio flux measurement in which concentrations are measured at two heights in the constant flux layer. Such an approach can provide a means to compute a measured deposition velocity of a surface-reactive substance as proportional to the ratio between the measured flux and the measured concentration difference.

## 4 Summary and recommendations

The number of chemical species simulated in chemical transport models (CTMs) has been increasing with increasing computer power. Among these, oVOCs and HCN are important groups of atmospheric pollutants for which dry deposition processes need to be treated as accurately as possible so that their inputs to ecosystems (noting that some oVOCs are organic nitrogen) and their roles in other atmospheric chemistry processes (e.g., formation of ozone and secondary organic aerosols) can be assessed. Earlier dry deposition schemes considered very few oVOCs and need to be extended for more species. Dry deposition of HCN was assumed to be negligible in some CTMs (e.g., Moussa et al., 2016). The present study first generated the effective Henry’s law constant and oxidizing capacity, the two key physical and chemical properties that are considered to control the dry deposition process (Wesely and Hicks, 2000), for 12 oVOC species and HCN. Two scaling factors for the non-stomatal resistance and one for the mesophyll resistance were applied to individual oVOCs and HCN for calculating their respective *V*_d_.

The modeled nighttime *V*_d_ agrees well with the measured data for most of the oVOCs, suggesting that the current non-stomatal parameterization scheme is a reasonable approach. The stomatal conductance for water vapor, with adjusted (reduced) *R*_s,min_, also agrees well with measured values. However, the modeled peak *V*_d_ values during daytime are only a fraction (0.2–0.5) of the measured values for some of the oVOCs, suggesting that fast atmospheric chemical processes likely contributed to the total measured fluxes. In practice, these additional fluxes during daytime can be modeled as non-stomatal uptake, and better model–measurement agreement can be obtained by adjusting the non-stomatal parameterization scheme (e.g., Müller et al., 2018; Paulot et al., 2018). However, using this approach will produce unreasonably high values for the solubility parameter and overpredict *V*_d_ during nighttime if the same non-stomatal formulas are used for both day and nighttime (as is the case in the existing schemes). More importantly, the high measured *V*_d_ values have only been observed at relatively few sites during very short periods (Karl et al., 2010; Nguyen et al., 2015). More evidence is needed to parameterize *V*_d_ for oVOCs to different land use categories over entire seasons. Until then, the conservative estimates of *V*_d_ such as modeled in this study are still recommended for use in CTMs. The model parameters chosen for *V*_d_ of these oVOCs provide the best-known representation of their respective physicochemical properties, and the modeled *V*_d_ values fall within the range of the low-end values of the available measurements.

Future field studies should focus on conducting flux measurements of oVOC compounds with the highest uncertainties, such as those that are most chemically reactive in the atmosphere or most rapidly taken up by wet surfaces. Additional measurements are also needed in different ecosystems to inform the representativeness of the high oVOC *V*_d_ reported by Nguyen et al. (2015) and Karl et al. (2010). Furthermore, concurrent chemical measurements of oxidants such as O_3_ and radicals are needed to quantify flux divergence due to fast within- and near-canopy chemical reactions. Future dry deposition schemes should include additional biochemical processes and species-dependent parameters for non-stomatal uptake, including enzymatic reactions (Karl et al., 2010), the octanol–air partitioning coefficients to account for cavity formation and polar intermolecular interactions with leaf surfaces and reservoirs (Nizzetto and Perlinger, 2012), and the enhancement and reduction effects due to soil and leaf moisture. Chemical processes within the canopy airspace could also be coupled with emission and deposition schemes to realistically simulate chemical fate and transport, including bidirectional fluxes of the reactive compounds discussed here and less reactive compounds such as methanol. Such an approach would require specification of chemical conditions within and near the canopy as well as in-canopy radiation and airflow. While more computationally intensive, the results presented here reinforce the need for such advanced models to explicitly resolve the non-stomatal processes contributing to the net atmosphere–biosphere exchange of reactive compounds. Above all, intercomparison studies should first be conducted for existing models that can handle oVOC dry deposition processes to quantify the magnitudes of uncertainties in the simulated *V*_d_ as well as the associated ambient concentrations and deposition fluxes.

## Supplementary Material

Supplement1

## Figures and Tables

**Table 1.** List of model parameters needed in the scheme of Zhang et al. (2003) to simulate the dry deposition velocity of additional oVOC species and HCN: *α* and *β* are scaling parameters for non-stomatal resistance, and *R*_m_ is mesophyll resistance.

Symbol	Name	Molecular weight (Da)	Scaling Parameters		*R*_m_ (sm^−1^)
			*α*	*β*	
HMHP	hydroxymethyl hydroperoxide	64	5	1	0
HAC	hydroxyacetone	74	1.5	1	0
PAA	peroxyacetic acid	76	2	1	0
HDC_4_	the C_4_ hydroxy dicarbonyl from IEPOX oxidation	102	1	0.2	0
DHC_4_	the C_4_ dihydroxy carbonyl from IEPOX oxidation	104	2	0.2	0
HPALD	isoprene hydroperoxy aldehydes	116	1.5	1	0
ISOPOOH/IEPOX^a^	isoprene hydroxyhydroperoxide and isoprene dihydroxyepoxide	118	5	0.2	0
PROPNN	propanone nitrate or propanal nitrate	119	1.5	1	0
ISOPN	isoprene hydroxy nitrates	147	1.5	1	0
MACN/MVKN^a^	methacrolein and methyl vinyl ketone hydroxy nitrate	149	1.5	1	0
INP	isoprene nitrooxy hydroperoxide	163	1.5	1	0
MTNP	monoterpene nitrooxy hydroperoxide	231	1.5	1	0
HCN	hydrogen cyanide	27	0	0.1	100
HCOOH^b^	formic acid	46	2	0.2	0

a Treated as one group of compounds in the field measurements due to instrument limitations and have the same parameter values in the model.

b The *β* value for HCOOH in Zhang et al. (2003) is 0.0, and here it is given as 0.2 to be consistent with other oVOC species (which would make no difference since the *α* value of 2 would dominate the non-stomatal resistance).

**Table 2.** Statistical results of the observed and modeled dry deposition velocity (*V*_d_) for oVOCs and HCN ( cm s^−1^)*.

Compound	All					Daytime				Nighttime		
	
	*N*	Obs	Mod	Mod-*R*_s,min_	*R*	*N*	Obs	Mod	Mod-*R*_s,min_	*N*	Obs	Mod
HMHP	247	1.66 (0.61)	0.69 (0.54)	0.75 (0.58)	0.63	85	3.42 (3.49)	1.05 (1.04)	1.19 (1.17)	128	0.33 (0.13)	0.37 (0.24)
HAC	245	0.84 (0.53)	0.41 (0.31)	0.49 (0.36)	0.61	84	1.21 (1.07)	0.65 (0.62)	0.81 (0.78)	128	0.44 (0.12)	0.21 (0.15)
PAA	243	1.08 (0.52)	0.46 (0.34)	0.53 (0.37)	0.74	85	2.18 (2.15)	0.71 (0.69)	0.86 (0.83)	128	0.28 (0.09)	0.24 (0.17)
HDC_4_	205	0.45 (0.22)	0.30 (0.20)	0.37 (0.23)	0.64	66	0.91 (0.78)	0.51 (0.49)	0.66 (0.65)	111	0.10 (0.06)	0.15 (0.10)
DHC_4_	247	0.42 (0.21)	0.41 (0.31)	0.47 (0.36)	0.61	85	0.92 (0.85)	0.63 (0.61)	0.76 (0.73)	128	0.08 (0.06)	0.22 (0.16)
HPALD	247	1.11 (0.46)	0.39 (0.29)	0.45 (0.34)	0.67	85	2.08 (2.17)	0.60 (0.58)	0.73 (0.70)	128	0.40 (0.10)	0.21 (0.15)
ISOPOOH/IEPOX	247	1.02 (0.49)	0.63 (0.48)	0.67 (0.52)	0.59	85	2.11 (2.06)	0.94 (0.94)	1.05 (1.05)	128	0.28 (0.09)	0.34 (0.23)
PROPNN	246	0.89 (0.43)	0.39 (0.29)	0.45 (0.33)	0.53	84	1.40 (1.38)	0.60 (0.58)	0.73 (0.70)	128	0.46 (0.13)	0.21 (0.15)
ISOPN	247	0.68 (0.39)	0.38 (0.28)	0.43 (0.33)	0.62	85	1.27 (1.29)	0.58 (0.57)	0.70 (0.67)	128	0.21 (0.09)	0.21 (0.15)
MACN/MVKN	246	0.65 (0.32)	0.38 (0.28)	0.43 (0.32)	0.57	84	1.19 (1.15)	0.58 (0.57)	0.70 (0.66)	128	0.22 (0.06)	0.21 (0.15)
INP	247	0.64 (0.46)	0.38 (0.28)	0.43 (0.33)	0.63	85	1.12 (1.17)	0.57 (0.56)	0.68 (0.65)	128	0.24 (0.10)	0.20 (0.15)
MTNP	246	0.33 (0.13)	0.36 (0.27)	0.40 (0.31)	0.54	84	0.55 (0.57)	0.54 (0.54)	0.64 (0.62)	128	0.16 (0.04)	0.20 (0.15)
HCN	234	0.13 (0.06)	0.17 (0.15)	0.22 (0.20)	0.77	84	0.26 (0.24)	0.33 (0.34)	0.43 (0.45)	117	0.03 (0.01)	0.03 (0.01)
HCOOH	244	0.47 (0.27)	0.46 (0.35)	0.54 (0.41)	0.52	83	0.82 (0.75)	0.72 (0.68)	0.91 (0.88)	127	0.20 (0.05)	0.23 (0.16)

* Note: *N* is the number of samples; *R* is the correlation coefficient between observation (Obs) and model simulation (Mod); “Mod-*R*_s,min_” refers to a sensitivity test in which *R*_s,min_ was reduced by 40%; daytime is 09:00–17:00 (local time) and nighttime is 20:00–06:00 (local time). Median values are provided in parentheses, following arithmetic mean values.
