# Supplementary material for: Extension of a gaseous dry deposition algorithm to oxidized volatile organic compounds and hydrogen cyanide for application in chemistry transport models
Source: J Adv Model Earth Syst. Author manuscript; Available in PMC 2022 Aug 16. (PMC8549847; doi:10.5194/gmd-14-5093-2021)
Supplement: Supplement1 [file NIHMS1735693-supplement-Supplement1.pdf]

Supplement of Geosci. Model Dev., 14, 5093–5105, 2021
https://doi.org/10.5194/gmd-14-5093-2021-supplement
© Author(s) 2021. CC BY 4.0 License.

Geoscientific Model Development
Open Access

Supplement of

Extension of a gaseous dry deposition algorithm to oxidized volatile organic compounds and hydrogen cyanide for application in chemistry transport models

Zhiyong Wu et al.

Correspondence to: Leiming Zhang (leiming.zhang@canada.ca)

The copyright of individual parts of the supplement might differ from the article licence.

Table S1. Henry's Law (*H*) and effective Henry's Law (*H**) values for oVOCs and HCN

Compound	*H* (M atm^{-1}) [a]	*H* for surrogate species if available (M atm^{-1}) [b]	*H* (M atm^{-1}) [c]	*H** (M atm^{-1}) [d]
HMHP	1.3×10^{6}	1.72×10^{6}	1.72×10^{6}	*H**=*H* [e]
HAC	2×10^{3}	7.80×10^{3}	7.80×10^{3}	*H**=*H*
PAA	5.2×10^{2}	8.41×10^{2}	8.41×10^{2}	Assuming same dissociation as acetic acid (K = 1.75×10^{-5} M); $H^* = 8.41 \times 10^2 \left\{1 + \frac{K}{[H^+]}\right\}$ =1.48×10^{5}
HDC$_4$	2×10^{3}	7.4×10^{1} (2,3-butanedione) 1.22×10^{2} (butanol)	1.22×10^{2}	*H**=*H*
DHC$_4$	2×10^{3}	2.1×10^{5} (1,2-butanediol)	2.1×10^{5}	*H**=*H*
HPALD	4×10^{4}	1.32×10^{-2} (isoprene), 6.28×10^{1} (tert-butyl hydroperoxide)	6.28×10^{1}	*H**=*H*
ISOPOOH	7×10^{7}	4.46×10^{5} (bis-(hydroxymethyl)-peroxide) 1.76×10^{6} (hydroxymethyl hydroperoxide)	1.76×10^{6}	*H**=*H*
IEPOX	7×10^{7}	1.93×10^{9} to 9.73×10^{10} [f]	1.93×10^{9} [g]	*H**=*H*
PROPNN	1×10^{4}	1.01×10^{3} (1-nitrooxy-2-propanone)	1.01×10^{3}	*H**=*H*
ISOPN	5×10^{3}	8.92×10^{3} (2-nitroxy-1-butanal) 3.65×10^{4} (5-nitrooxy-2-pentanol)	3.65×10^{4}	*H**=*H*
MACN	6×10^{3}	3.65×10^{4} (5-nitrooxy-2-pentanol) 9.02×10^{3} (2-nitrooxy-1-butanol) 1.01×10^{3} (1-nitrooxy-2-propanone)	3.65×10^{4}	*H**=*H*
MVKN	6×10^{3}	3.65×10^{4} (5-nitrooxy-2-pentanol) 9.02×10^{3} (2-nitrooxy-1-butanol) 1.01×10^{3} (1-nitrooxy-2-propanone)	3.65×10^{4}	*H**=*H*
INP	5×10^{3}	6.28×10^{1} (tert-butyl hydroperoxide) 3.65×10^{4} (5-nitroxy-2-pentanal)	3.65×10^{4}	*H**=*H*
MTNP	1×10^{3}	2.13×10^{4} (1-methyl-1-phenylethylhydroperoxide)	2.13×10^{4}	*H**=*H*
HCN	1×10^{1}	1.72×10^{1}	1.72×10^{1}	HCN (aq) ⇔ H^{+} + CN^{-} (K = 6.2×10^{-10} M) $H^* = 1.72 \times 10^1 \left\{1 + \frac{K}{[H^+]}\right\}$ =1.73×10^{1}

[a] Data listed in Nguyen et al. (2015) which is mostly based on Sander (1999).
[b] Data from Sander (2015); The unit conversion was taken using 1 mol m^{-3} Pa^{-1} = 101.325 M atm^{-1}.
[c] Larger value of the surrogates is chosen.
[d] $H^* = H\left(1 + \frac{K}{[H^+]}\right)$, [H^{+}] = 1×10^{-7} M.
[e] Note that pKa (= -log$_{10}$Ka) for alcohols ranges from 15.5 for methanol to 18.3 for 3-pentanol. If methanol is assumed, then Ka = 3.16×10-16, so very small contribution to *H* law and this contribution decreases with increasing molecular mass.
[f] Chemical structures for these compounds follow Chan et al. (2010).
[g] Note that Sander (2015) noted a higher degree of uncertainty associated with this value.

Table S2. Oxidizing capacities for oVOCs and HCN calculated using the methods described in Reid et al. (1987)

Compound	ΔG_f for species (J/mol) [a]	Example Redox Reaction. W Reactant + X H(+) + X e^- → Y CH_4(g) + Z H_2O $\Delta G_{f,CH4} = -5.087\times10^4$, $\Delta G_{f,H2O} = -2.288\times10^5$, $\Delta G_{f,N2} = 0$	Resulting ΔG_f for the reaction (J/mol)	pe^0 [b]	n_H [c]	$pe^0(W)$ [d]
HMHP	-3.9078×10^5	HMHP + 6 H(+) + 6 e^- → CH_4(g) + 3 H_2O	-3.465×10^5	10.134	6	3.13
HAC	-3.4524×10^5	HAC + 10 H(+) + 10 e^- → 3 CH_4(g) + 2 H_2O	-2.650×10^5	4.650	10	-2.35
PAA	-3.8023×10^5	PAA + 10 H(+) + 10 e^- → 2 CH_4(g) + 3 H_2O	-4.079×10^5	7.158	10	0.16
HDC_4	-4.6574×10^5	HDC4 + 16 H(+) + 16 e^- → 4 CH_4(g) + 3 H_2O	-4.241×10^5	4.652	16	-2.35
DHC_4	-4.7608×10^5	DHC4 + 12 H(+) + 12 e^- → 4 CH_4(g) + 3 H_2O	-4.138×10^5	6.051	12	-0.95
HPALD	-2.4813×10^5	HPALD + 18 H (+) + 18 e^- → 5 CH_4(g) + 3 H_2O	-6.926×10^5	6.753	18	-.025
ISOPOOH	-2.8025×10^5	ISOPOOH + 16 H(+) + 16 e^- → 5 CH_4(g) + 3 H_2O	-6.605×10^5	7.244	16	0.24
IEPOX	-3.7487×10^5	IEPOX + 16 H(+) + 16 e^- → 5 CH_4(g) + 3 H_2O	-5.659×10^5	6.207	16	-0.79
PROPNN	-2.7787×10^5	PROPNN + 15 H(+) + 15 e^- → 3 CH_4(g) + 4 H_2O	-7.899×10^5	9.242	15	2.24
ISOPN	-1.9208×10^5	DHC4 + 19 H(+) + 19 e^- → 5 CH_4(g) + 4 H_2O	-9.775×10^5	9.028	19	2.03
MACN	-3.7405×10^5	MACN + 19 H(+) + 19 e^- → 4 CH_4(g) + 5 H_2O	-9.561×10^5	8.831	19	1.83
MVKN	-4.0871×10^5	MVKN + 19 H(+) + 19 e^- → 4 CH_4(g) + 5 H_2O	-9.561×10^5	8.831	19	1.83
INP	-3.0226×10^5	INP + 21 H(+) + 21 e^- → 5 CH_4(g) + 5 H_2O	-1.096×10^5	9.160	21	2.16
MTNP	-2.4833×10^5	MTNP + 34 H(+) + 34 e^- → 10 CH_4(g) + 5 H_2O	-1.404×10^6	7.249	34	0.25
HCN	$+1.202\times10^5$	2 HCN + 6 H(+) + 6 e- → 2 CH_4(g) + N_2(g)	-3.421×10^5	10.007	6	3.01

[a] The ΔG_f value for HCN was based on observations presented in Reid et al. (1987). Remaining values were calculated using the Joback group method as described in Reid et al. (1987).
[b] pe^0 is the logarithm of the electron activity of the reaction, $pe^0 = -\Delta G_f/(2.3RT)$, where R = 8.314 J mol^{-1} K^{-1}, and T = temperature (K), defined as 298K here.
[c] n_H is the number of hydrogen atoms in the redox reaction.
[d] $pe^0(W)$ is the value of pe^0 at a pH of 7; $pe^0(W) = pe^0 - n_H 7$.

Table S3. Mean and median values of the observation-based residual conductance ($G_{residual}$) and the modeled non-stomatal conductance (G_{ns}) under different conditions (cm s^{-1}).

Compound	Nighttime dry surface				Nighttime wet surface				Daytime dry surface			
	observation-based $G_{residual}$		modeled G_{ns}		observation-based $G_{residual}$		modeled G_{ns}		observation-based $G_{residual}$		modeled G_{ns}	
	median	mean	median	mean	median	mean	median	mean	median	mean	median	mean
HMHP	0.10	0.13	0.42	0.48	0.29	0.30	1.02	1.03	7.17	8.73	1.05	1.02
HAC	0.09	0.11	0.15	0.18	0.20	0.32	0.36	0.37	0.75	0.94	0.44	0.43
PAA	0.13	0.16	0.19	0.22	0.23	0.25	0.46	0.46	3.48	4.09	0.55	0.55
HDC_4	0.08	0.09	0.08	0.10	0.46	0.54	0.21	0.22	0.72	0.76	0.30	0.29
DHC_4	0.11	0.13	0.16	0.19	0.18	0.21	0.40	0.40	0.61	0.70	0.47	0.45
HPALD	0.08	0.11	0.15	0.18	0.22	0.32	0.36	0.37	3.28	3.53	0.44	0.43
ISOPOOH/IEPOX	0.10	0.11	0.39	0.44	0.36	0.31	0.97	0.98	3.41	3.70	0.99	0.96
PROPNN	0.14	0.16	0.15	0.18	0.23	0.36	0.36	0.37	1.66	1.70	0.44	0.43
ISOPN	0.16	0.18	0.15	0.18	0.34	0.45	0.36	0.37	1.40	1.49	0.44	0.43
MACN/MVKN	0.14	0.18	0.15	0.18	0.49	0.62	0.36	0.37	1.21	1.26	0.44	0.43
INP	0.09	0.10	0.15	0.18	0.46	0.46	0.36	0.37	1.24	1.25	0.44	0.43
MTNP	0.08	0.08	0.15	0.18	0.31	0.39	0.36	0.37	0.50	0.62	0.44	0.43
HCN	0.01	0.01	0.01	0.01	0.04	0.04	0.01	0.01	0.04	0.04	0.04	0.04
HCOOH	0.12	0.12	0.16	0.18	0.19	0.25	0.40	0.41	0.46	0.53	0.47	0.45

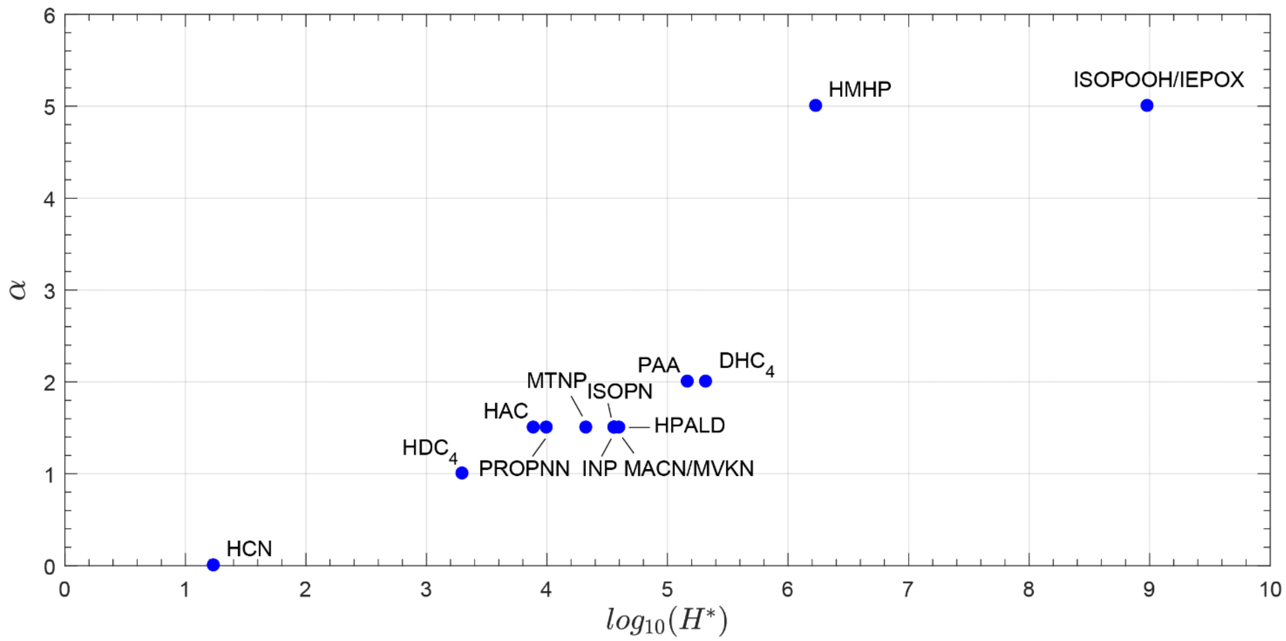

Figure S1. The chosen model parameter (α) versus literature reported maximum effective Henry's Law (H*) values for oVOCs and HCN considered in the present study.

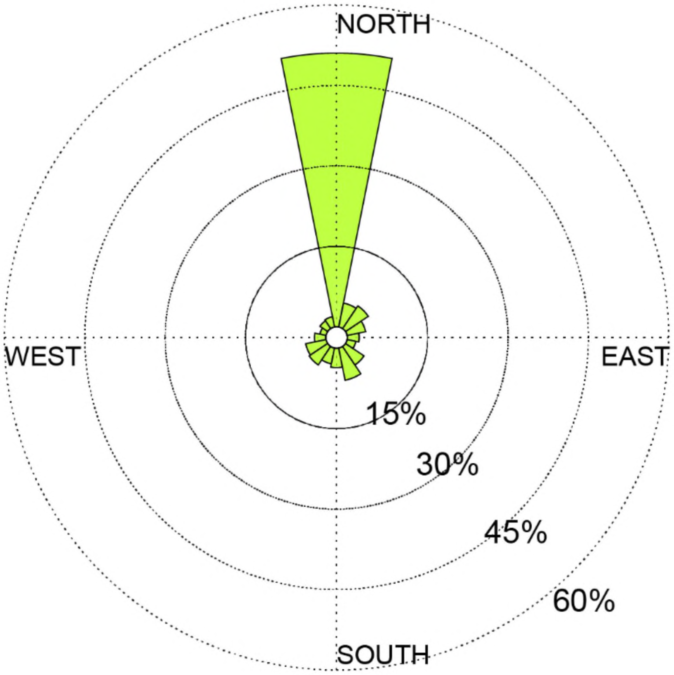

Figure S2. Windrose at the CTR site during the study period.

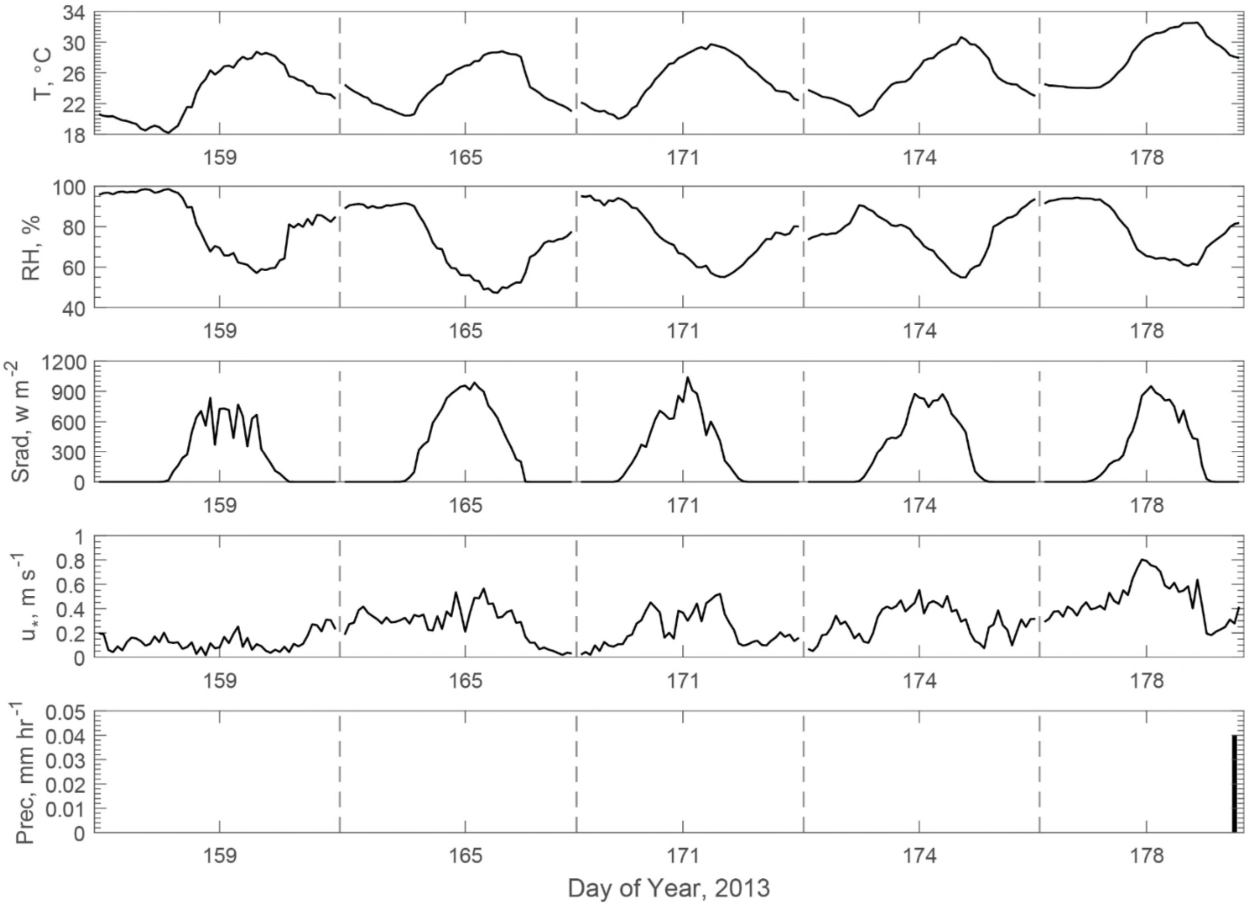

Figure S3. Meteorological parameters at the CTR site during the study period, including temperature (*T*), relative humidity (*RH*), solar radiation (*Srad*), friction velocity (u_*), and precipitation (*Prec*).

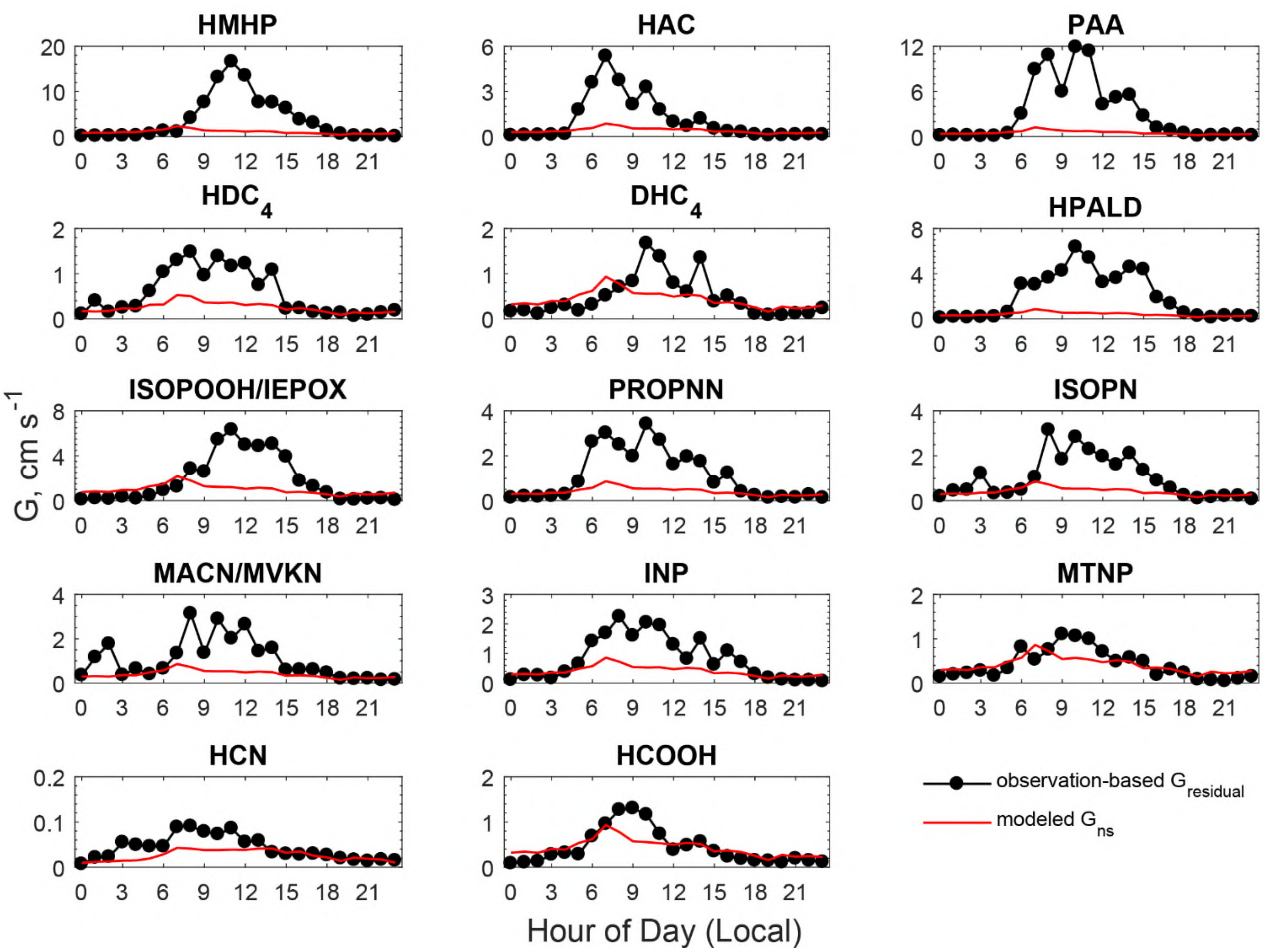

Figure S4. Comparison of averaged diel variations of the observation-based residual conductance ($G_{residual}$) and the modeled non-stomatal conductance (G_{ns}) of oVOCs and HCN.

References:

Chan, M.N., Surratt, J.D., Claeys, M., Edgerton, E.S., Tanner, R.L., Shaw, S.L., Zheng, M., Knipping, E.M., Eddingsaas, N.C., Wennberg, P.O. Seinfeld, J.H., 2010. Characterization and quantification of isoprene-derived epoxydiols in ambient aerosol in the southeastern United States. *Environmental Science & Technology*, 44(12), 4590-4596.

Nguyen, T.B., Crounse, J.D., Teng, A.P., Clair, J.M.S., Paulot, F., Wolfe, G.M., Wennberg, P.O., 2015. Rapid deposition of oxidized biogenic compounds to a temperate forest. *Proceedings of the National Academy of Sciences*, 112, E392-E401.

Reid, R.C., Prausnitz, J.M., Poling, B.E., 1987. The properties of gases and liquids, 4th Edition. McGraw-Hill, Inc., New York, 741 pp.

Sander, R., 1999. Compilation of Henry's Law constants for inorganic and organic species of potential importance in environmental chemistry. Max-Planck Institute of Chemistry, Air Chemistry Dept., Mainz, Germany.

Sander, R., 2015. Compilation of Henry's law constants (version 4.0) for water as solvent. *Atmospheric Chemistry & Physics*, 15(8), 4399-4981.
